# The Young Stethescopist, or the Student’s Aid to Auscultation

**Published:** 1848-06

**Authors:** 


Art. VII.—*The Young Stethoscopist; or the Student's aid to Auscultation.* By Henry J. Bowditch, M. D., one of the Physicians of the Massachusetts General Hospital. Second edition. New York; Samuel S. & William Wood. pp. 303.

We are glad to see a second edition of this little work, and we trust that every young student of auscultation will furnish himself with a copy. It is an excellent introduction to the larger works. Dr. Bowditch seems to have right notions of the value of physical signs, and he does not fail to impress his views on the student.

"Amidst the niceties of our physical examinations we are apt to neglect the rational signs. The truth is, that he who scoffs at either must necessarily be a child in the diagnosis of not a few diseases; and he who cultivates both with the clear, keen-sighted eye of a true observer, and then notes their mutual relations, is the truly wise physician. Both categories of signs are useful, each in its own sphere; and neither should be allowed to predominate; neither should be neglected."

This is well and timely said, and breathes the spirit in which the book has been produced. C.

